# Lipids in a Nutshell: Quick Determination of Lipid Content in Hazelnuts with NIR Spectroscopy

**DOI:** 10.3390/foods12010034

**Published:** 2022-12-22

**Authors:** Elena Cazzaniga, Nicola Cavallini, Alessandro Giraudo, Gentian Gavoci, Francesco Geobaldo, Mattia Pariani, Daniela Ghirardello, Giuseppe Zeppa, Francesco Savorani

**Affiliations:** 1 Department of Applied Science and Technology, Politecnico di Torino, Corso Duca degli Abruzzi 24, 10129 Turin, Italy; 2 Department of Agricultural, Forest and Food Sciences, University of Turin, Via Leonardo da Vinci 44, Grugliasco, 10095 Turin, Italy

**Keywords:** food, hazelnuts, chemometrics, NIR calibration

## Abstract

Hazelnuts (*Corylus avellana* L.) are among the most consumed dry fruits all over the world. Their commercial quality is defined, above all, by origin and dimension, as well as by lipid content. Evaluation of this parameter is currently performed with chemical methods, which are expensive, time consuming, and complex. In the present work, the near-infrared (NIR) spectroscopy, using both a benchtop research spectrometer and a retail handheld instrument, was evaluated in comparison with the traditional chemical approach. The lipid content of hazelnuts from different growing regions of origin (Italy, Chile, Turkey, Georgia, and Azerbaijan) was determined with two NIR instruments: a benchtop FT-NIR spectrometer (Multi Purpose Analyser—MPA, by Bruker), equipped with an integrating sphere and an optic fibre probe, and the pocket-sized, battery-powered SCiO molecular sensor (by Consumer Physics). The Randall/Soxtec method was used as the reference measurement of total lipid content. The collected NIR spectra were inspected through multivariate data analysis. First, a Principal Component Analysis (PCA) model was built to explore the information contained in the spectral datasets. Then, a Partial Least Square (PLS) regression model was developed to predict the percentage of lipid content. PCA showed samples distributions that could be linked to their total crude fat content determined with the Randall/Soxtec method, confirming that a trend related to the lipid content could be detected in the spectral data, based on their chemical profiles. PLS models performed better with the MPA instrument than SCiO, with the highest R^2^ of prediction (R^2^_PRED_ = 0.897) achieved by MPA probe, while this parameter for SCiO was much lower (R^2^_PRED_ = 0.550). Further analyses are necessary to evaluate if more acquisitions may lead to better performances when using the SCiO portable spectrometer.

## 1. Introduction

Hazelnuts (*Corylus avellana* L.) are largely farmed and exported worldwide, with a production volume of over one billion tons in 2020 [1]. Concerning the world production, Turkey is the main producer, covering approximately 70% of the total production. Italy is the following, with a share close to 20% of the total production. Other major producer countries are Azerbaijan, Georgia, and USA [2]. Among tree nuts, hazelnuts show high nutritional and nutraceutical properties, mainly due to their fat content (approximately 60% in weight), characterized by a beneficial fatty acids composition, most of which are unsaturated fatty acids (mainly oleic but also linoleic and linolenic acids) [3]. Other beneficial macronutrients contributing to the healthy properties of hazelnut are proteins, which provide near 25% of energy [4], as well as carbohydrates and dietary fibres. Significant amounts of essential micronutrients and non-nutritive components are present among the constituents of hazelnuts, including vitamins (e.g., tocopherols, valuable antioxidant vitamins), healthy minerals (such as calcium, magnesium, and potassium), phytosterols, and polyphenols [5]. The latter, synthesized by the plant to preserve the reproductive potential of the seed, protecting the germ from oxidative stress, can play a key role in human health if regularly consumed, as they are considered as potential opponents to cancer and diabetes, due to their ability to deactivate free radicals [6].

The lipid fraction is quantitatively the most abundant constituent of hazelnuts, and therefore, the most significant in terms of energy intake, organoleptic quality, and storability [4]. While, in some cases, the high hazelnut lipid content could be a positive and desired factor (e.g., for hazelnut oil production), others, such as the excessive calorie intake, the increased in susceptibility to lipid oxidation, or, from a technological point of view, the problem of oil migration in hazelnut paste/chocolate, could require an opposite choice. For these reasons, total fat content is one relevant trait, among quality attributes, considered by hazelnuts processors and manufacturers [7]. Many works have investigated the factors affecting the content and composition of hazelnut fats, and it is well documented that variety, geographical origin [1,8,9], harvest technique, and harvest year are key factors in the synthesis and accumulation of lipids in hazelnut kernels [7,10,11,12]. Recent studies also demonstrated the possibility of discerning, among different geographical production areas, using metabolomic approaches [13,14], and a study by Tüfekci and Karataş [2], based on gas chromatography analysis, has shown that the differences in fatty acid composition can be used for discriminating the geographical origin. However, the most used analytical techniques to determine and quantify the fatty acid composition, at laboratory scale, are generally time-consuming and involve expensive instrumentation, which also requires high scientific expertise.

Fourier transform near-infrared (FT-NIR) spectroscopy represents a rapid and cost-effective tool to inspect the properties of food [15]. This technique prevents the use of chemicals and potentially dangerous solvents by reducing, if not by completely avoiding, any sample pre-treatments, as well as by allowing preservation and recovery after the analysis of the analysed specimen, making it a non-destructive technique. NIR spectroscopy is generally coupled with the tools of chemometrics, i.e., multivariate data analysis approaches, for efficiently extracting information from large and complex data [16], and it uses them to explore the chemical composition of food products [17] or to prevent food fraud [18]. NIR spectroscopy combined with chemometrics was used for the detection of flawed kernels and to predict fat primary oxidation [7], but it was also used with the aim of discriminating different hazelnut varieties [19,20].

The aim of this study was to evaluate the use of NIR spectroscopy to perform the quantification of total lipids, thus potentially replacing the conventional Soxhlet extraction methods, which are expensive, time consuming, and involve dangerous and non-ecofriendly solvents. The performances of three NIR techniques (a portable instrument and a benchtop instrument operating in two acquisition modes) were assessed, proving that NIR spectroscopy, coupled with multivariate data analysis tools (i.e., Chemometrics), allows for rapid analyses performed in a non-destructive way directly on the ground samples, resulting in regression models able to predict the content of lipids in the hazelnuts.

## 2. Materials and Methods

### 2.1. Hazelnut Samples

A total of 56 samples of raw hazelnuts from different countries (Italy, Turkey, Azerbaijan, Georgia, and Chile) were analysed. Each sample was ground with a Retsch ZM200 grinder (Retsch Gmbh, Haan, Germany) and sieved using a vibratory sieve shaker BA 200N (CISA Sieving Technologies, Lliçà de Vall, Barcelona, Spain). Then, the particles of sizes in the range 250–500 µm were selected. All samples were stored in hermetically closed polyethylene bags at –20 °C and allowed to warm up to room temperature (~20 °C) before analysis.

### 2.2. Lipid Extraction—Randall/Soxtec Extraction

To reduce the volume of organic solvent used and the time extraction [21], the crude fat content of the samples was determined according to the Randall/Soxtec modification of the standard Soxhlet extraction method (AOAC 948.22) [22]. The hot solvent extraction process was carried out with a SER 148 Solvent Extractor (Velp Scientifica Srl, Usmate Velate (MB), Italy) equipped with three Soxhlet posts. In each Soxhlet post, 5 ± 0.001 g of hazelnuts powder was extracted with 99% n-hexane, analytical grade (Sigma-Aldrich, Milan, Italy), for a total of 120 min. The extraction process involved three semi-automated steps. During the first step, the thimbles containing the sample were immersed in the boiling solvent (60 mL, 130 °C, 60 min); then, the level of the solvent was lowered below the extraction thimbles. The second step (washing step—60 min) allowed the continuous flow of condensed solvent over the sample and through the thimble, completing the solvent extraction. During the last step (30 min), as much solvent as possible was distilled and recovered from extraction cups until apparent dryness. Finally, the extraction cups, including the extracts, were dried at 105 °C for 1 h, cooled in a desiccator to room temperature, and weighed for the extract percentage calculation.

The results were then reported as total grams of extracted lipids, with their respective percentages related to the weight of ground hazelnuts. For each hazelnut sample, three replicates were extracted simultaneously.

### 2.3. NIR Instrumentation and Spectra Acquisitions

All NIR analyses were performed using two spectrometers with different spectral ranges and resolutions: a benchtop Fourier transform-NIR (FT-NIR) spectrometer (Multi-Purpose Analyser—MPA, Bruker Optics, Ettlingen, Germany) equipped with an integrating sphere and an optical fibre reflectance probe, as well as the SCiO Pocket molecular sensor (v1.2, Consumer Physics Inc., Tel Aviv, Israel).

The instrumental settings for the MPA operated in sphere mode were: 800–2780 nm (12,500–3600 cm^–1^) spectral range, 8 cm^–1^ optical resolution, and 10 kHz scanner velocity. A sample holder of 9 cm of diameter, equipped with a quartz window on the bottom, was used and carefully cleaned in between each sample acquisition. Regarding the MPA operated in optical fibre probe mode the instrumental settings were: 800–2500 nm (12,500–4000 cm^–1^) spectral range, 16 cm^–1^ optical resolution and 20 kHz (probe) scanner velocity. MPA probe measurements were performed by gently inserting the probe in the ground sample kept in its storage plastic bag, paying attention to not touch or place the NIR ray too close to the plastic material. In both MPA acquisition modes, 64 scans for both sample and background acquisition were collected, resulting in one individual averaged spectrum for each sample. Background scans were performed using the instrument’s internal reference standard. The Opus software (v6.5, Bruker Optics, Ettlingen, Germany) was used for instrumental control and for spectra acquisition.

The instrumental settings for the SCiO device are non-customizable and are fixed as follows: 740–1070 nm (13,510–9340 cm^–1^) spectral range, 10 cm^–1^ resolution, and time scan of approximately 5 s. Spectral scans were managed through the SCiO smartphone app (The Lab, version 2.5.3), which allows for controlling the sensor via Bluetooth connection and also uploads all acquired data on the Consumer Physics Cloud database, which is accessible via browser to inspect and download the raw data. The spectral measurements were done by gently placing the SCiO instrument on top of the sample and subsequently moving to other locations of the same sample mass to account for possible differences due to the uneven surface of the ground sample.

All NIR analyses were performed in reflectance mode. For each specimen, three spectra were collected as replicates with the benchtop MPA instrument, while six replicates were acquired with the SCiO portable device. An average spectrum was then calculated from the replicates after proper replicate quality evaluation. There was one average spectrum that was therefore obtained for each individual sample, and all further data analysis steps were done on the averaged spectra.

### 2.4. Multivariate Data Analysis

The raw NIR spectra were analysed under MATLAB environment (R2020a, The Mathworks Inc., Natick, MA, USA). Firstly, the MPA spectra needed to be cut at the extremities, to remove areas that were particularly noisy. The obtained range was 1117–2561 nm for the MPA operating in sphere mode and 1121–2254 nm for the MPA operating in probe mode. Such correction was not necessary for the SCiO spectra.

Furthermore, all datasets were pre-processed with Standard Normal Variate (SNV), [23] with the purpose of eliminating artefacts and correcting nonlinear behaviours, due to potential scattering effects originating from the granular nature of the samples. Mean-centring was also applied prior to any multivariate data analysis.

#### 2.4.1. Exploratory Data Analysis

Exploratory data analysis was performed using Principal Component Analysis (PCA, [24,25]), with the aim of obtaining information about the data quality, the presence of possible outliers and/or extreme samples, and, more importantly, to reveal the potential presence of a trend related to the lipid content, prior to the construction of the regression model. The first two PCs were considered the most informative, as the explained variance was equal or above 90% for all three different acquisition methods.

#### 2.4.2. Regression Models

The first step in the construction of the Partial Least Squares (PLS, [26,27]) model was splitting the spectral dataset into a training and a test set, with the test set consisting of 33% of the total number of samples: the resulting datasets for the three techniques consisted of 38 samples in calibration and 18 samples in the test set. This was done using the Duplex algorithm [28], which allows homogeneous sampling of the initial and complete dataset. Then, PLS was used for building the regression models to predict the lipid content of hazelnuts, expressed in percentage, as obtained with the Randall/Soxtec extraction method (Section 2.2). The regression performances were evaluated in terms of the coefficient of determination (R^2^), the root mean squared error (RMSE), and the Ratio of Prediction to Deviation (RPD, [29,30]). Regarding the models practical applicability based on the RPD values, we refer to the framework described by Williams [30]. All RPD interpretation efforts reported in the present work are referred to the RPD values related to samples of forages, feeds, and soils [30], which are described as “materials that are more complicated in terms of their physical nature”, which the authors considered the most correct term of comparison for the samples under examination."

## 3. Results and Discussion

**Figure 1:**
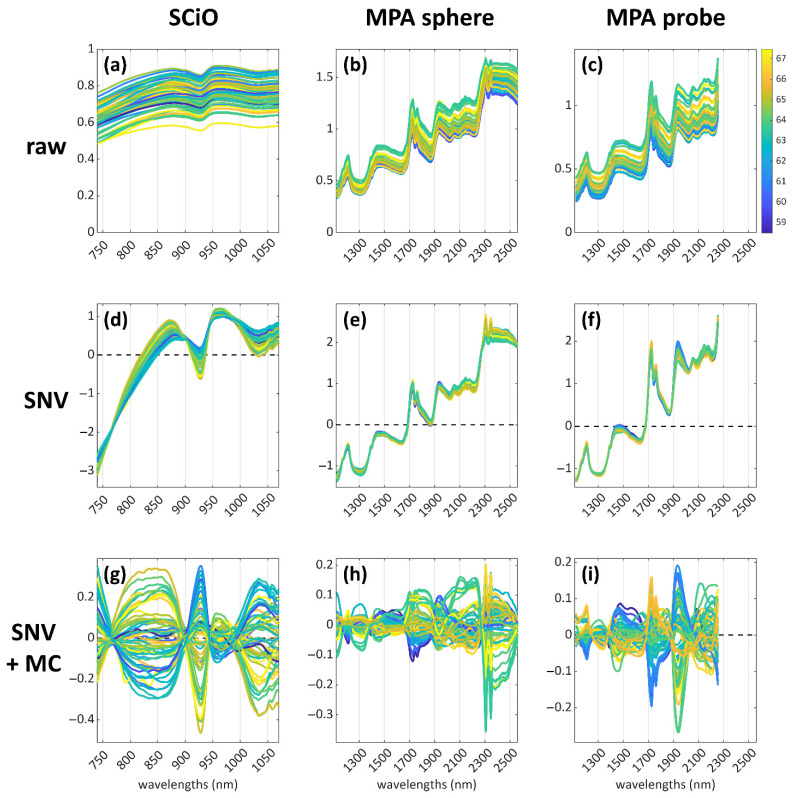
Visual representation of the data-preprocessing pipeline, declined, according to the three datasets (from top to bottom along each column): raw NIR spectra of the hazelnut samples (**a**–**c**); spectra preprocessed with Standard Normal Variate (SNV) (**d**–**f**); spectra preprocessed with SNV + mean centering (MC) (**g**–**i**).

To provide a clear description of the data analysis pipeline, a visual representation of the data preprocessing steps, declined according to the three datasets, is depicted in Figure 1. In the first row, the spectra with no pre-treatment (Figure 1a–c) are reported, followed by the spectra, preprocessed with SNV (Figure 1d–f), in the central row, and by the spectra with SNV + mean centre preprocessing (Figure 1g–i) in the bottom row. The samples are coloured according to their lipid content: blue corresponds to lower lipid content assessed by Randall/Soxtec extraction, while yellow corresponds to higher lipid content.

As stated in Section 2.4, the selected preprocessing approach was SNV followed by mean centre: no other preprocessing methods were tested due to the need of keeping the description of the results, obtained from three datasets, as clear and simple as possible. We would like to stress that this study is intended as a proof-of-concept with the aim of further improving it at the industrial hazelnut processing plant.

### 3.1. Exploratory Analysis Results

**Figure 2:**
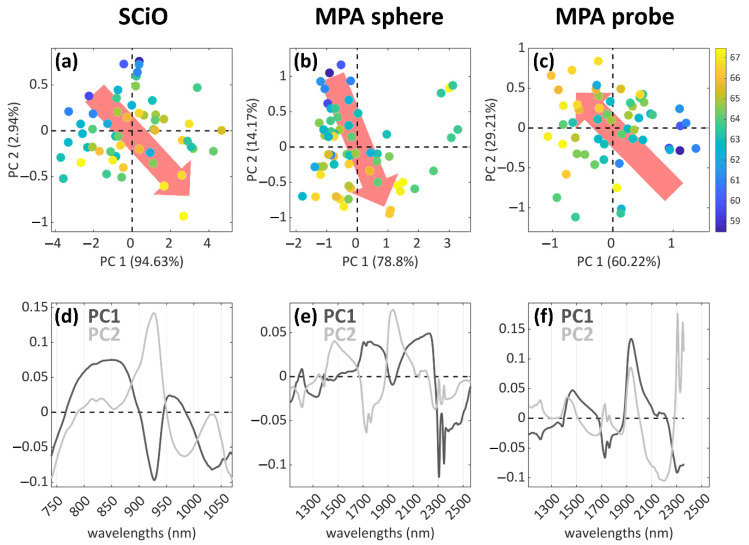
PCA results for the three analytical techniques of the study. The score plots are shown in the first row (**a**–**c**), with the samples coloured according to the lipid content and the content trends highlighted by the red arrows (from low to high). In the second row (**d**–**f**) the corresponding loadings plot are reported, showing the variables that are important for each PC reported in (**a**–**c**).

Exploratory data analysis was performed to obtain preliminary information concerning the presence of a trend among the samples, due to the lipid content. Results are shown in Figure 2: the most informative combinations of principal components are reported for each dataset (Figure 2a–c) together with the respective loadings (Figure 2d–f). The samples are coloured according to their lipid content, and a trend in the distribution of the samples can be observed: the scores follow the directions described by the red arrows, from lower to higher contents of lipids.

These exploratory analyses suggest that a trend related to the lipid content can be detected in the spectral data, according to their chemical profile. Based on these considerations, it was decided to proceed with a further modelling step aimed at predicting the content in fatty acids, directly from the spectral information, by means of PLS regression.

### 3.2. PLS Regression Results

**Figure 3:**
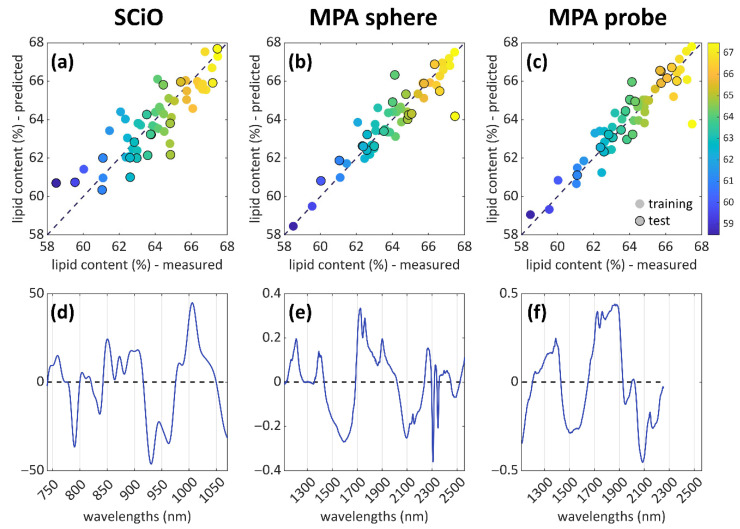
PLS results for the three analytical techniques of the study. The prediction plots are shown in the first row (**a**–**c**), with the samples coloured according to the lipid content. The black-bordered markers correspond to the test set (predicted samples). In the second row (**d**–**f**), the corresponding regression vectors are reported, showing the variables that are important for each PLS model.

One PLS regression model was built for each properly preprocessed spectral dataset (i.e., MPA sphere, MPA probe, SCiO), with model-specific training and test sets for model building and validation. The regression parameters are summarised in Table 1, where the models’ complexities (number of latent variables, LVs), the coefficients of determination (R^2^), the root mean squared errors (RMSEs), and the RPD values are reported. Both calibration and cross-validation (CV) values are reported in this table: the parameters referring to CV are generally of major interest, as the CV procedure better elucidates the robustness of the information contained in the data, and consequently, the evaluation of the model performances is more reliable. Moreover, a visual representation of the predicted lipid content values (expressed in percentage) plotted against the measured ones is provided in Figure 3a–c: together, with the regression parameters of Table 1 and the regression vectors of Figure 3d–f, a complete overview of the models is therefore given.

According to these results, the MPA sphere showed the best results for both calibration and validation of the regression model, with a value of R^2^_CV_ = 0.903, which was higher than those obtained with MPA probe and SCiO. From the point of view of the model’s error (the RMSEs), the MPA sphere in validation was also the lowest, with RMSE_CV_ = 0.645, confirming that MPA sphere models seemed to be more robust than the others. Despite this, even better results were achieved with the MPA probe concerning the prediction step, with R^2^_PRED_ = 0.897 and RMSE_PRED_ = 0.712, suggesting that this technique could be performing better in correctly predicting the lipid content of hazelnuts when compared to the other NIR techniques used for this purpose.

The SCiO results were globally worse than those obtained with the benchtop MPA instrument, especially regarding the values of the coefficients of determination, with R^2^_CV_ = 0.461 and R^2^_PRED_ = 0.550, which resulted in much worse values than those obtained with the MPA instrument. This could be due to the limited spectral range of acquisition of this portable instrument, which can represent a limit if compared to a benchtop spectrometer. In fact, the SCiO spectral absorption range only contains the last overtones and combination bands of the NIR interval, while the larger acquisition range of MPA can detect more signals than SCiO. Moreover, the number of samples probably also represented a limitation to the performances of this instrument. Despite this, the results obtained with the SCiO were interesting in the perspective of in situ analyses: even if this technique is less accurate, it could lead to reliable models if the number of samples employed to build the calibration model is increased.

From the point of view of the RPD values, the best model, even if failing in prediction, proved to be the MPA sphere acquisition mode, with values above 3 for the calibration and CV models. These results put the MPA sphere model in the “screening/quality control” RPD bracket according to Williams [30], even if the prediction RPD value of this model is very poor according to the same RPD scheme. The MPA probe model can be placed into the “rough screening” RPD bracket, and this is consistent with the interpretation of the other regression parameters, as discussed before: the model shows lower performances if compared to the MPA sphere acquisition mode; therefore, the suggested applicability by Williams [30] is also less reliable (screening/quality control vs. rough screening). The SCiO model once again proved to be less reliable, showing very poor performances from the RPD point of view, especially when considering the CV figure, which places this model into the Williams [30] bracket of “not recommended” to be used. It is important to consider that the aim of the study was to assess the performances of the different instruments and acquisition modes, and the rather limited number of samples already showed some limitations on the models’ performances when inspected from the point of view of the traditional chemometric regression figures (coefficient of determination and RMSE).

To identify and interpret the most influent NIR signals, the regression vectors of each model were inspected (Figure 3d–f). Lipids in hazelnuts represent about 60% of the total amount of components, so the signals of the regression coefficients can be interpreted, mostly, related to the lipids signals. The information found in literature concerning the determination of fatty acids in hazelnuts shows that the main fatty acids analysed in this substrate are palmitic, stearic, oleic, and linoleic acids, with a percentage of unsaturated acids above 90% [31].

The interpretation of the regression coefficients is resumed in Table 2. Aliphatic peaks can be found in the region 1700–1900 nm, and they are related to the first overtone, C–H, stretching [32,33]. These peaks fall within the MPA spectral range, as the SCiO only covers the wavelengths between 740 and 1070 nm. In particular, the signals referred to this chemical group are the two peaks in the range of 1730–1760 nm, which appear well defined for the MPA sphere, while the MPA probe shows a partial overlap with the band at ~1900 nm, which is ascribable to O–H deformation and stretching combination bands. Another strong signal is a doublet at 2300–2340 nm, which could be related to C–H stretching and deformation combinations of CH_2_ of lipids. As the largest part of fatty acids in hazelnuts is composed by unsaturated fatty acids, a strong band at 2100 nm, related to the C=C stretching, can be noticed. Other intense signals are the C–H second overtone at 1214 nm of CH_2_, and CH_2_ stretching at 1390 nm.

The SCiO spectral range showed the vibration associated with the third overtone of the C–H stretch of lipids at ~920 nm [32]. Other strong signals detected by this instrument can be referred to with O–H stretching second overtones, in the range between 960–1050 nm [33]. This instrument can detect the signals related to the absorption of fatty acids, in accordance with the aim of this study. This information was used in the construction of the model, even if the regression parameters underline that the obtained model is not robust enough to be used for reliable predictions. This issue might be solved by considering a larger number of samples to be included in the model.

## 4. Conclusions

The evaluation of the chemical composition, and particularly of the lipid content of hazelnuts, is very important for their industrial use and economic evaluation. The determination of crude fat content is currently performed with Soxhlet extraction methods that are expensive and time consuming. In this work, the use of NIR spectroscopy was evaluated for the determination of total lipids in hazelnuts, similarly to what has been performed for other products, such as milk and seeds. NIR spectroscopy has many advantages if compared to the most known and traditionally used analytical techniques, as it allows for saving time in both the pre-treatment and operative steps, while at the same time, also being solvent-free. Moreover, with NIR spectroscopy the sample can be completely recovered after the analysis, which means that this technique is fully non-destructive.

There were two different NIR instruments used in this study: the portable SCiO sensor and the benchtop MPA, operating in sphere and probe modes. The obtained results highlighted that the information contained in the spectra, extracted and analysed with the tools of chemometrics, could be usefully exploited in the construction of rather robust regression models that proved to be able to predict the lipid content of hazelnut. In particular, the best results were obtained using the MPA probe, as the regression figures, in prediction, were the best if compared to the other techniques. The MPA sphere showed slightly worse performances in prediction, while the cross-validation step suggested that this model could be the most robust, with R^2^_CV_ = 0.903, RMSE_CV_ = 0.645, and RPD_CV_ = 3.254. The regression parameters of the SCiO portable molecular sensor were, in general, lower than MPA’s, suggesting that the model was not robust enough to obtain reliable results when applied to the analyses of new samples.

Additional analyses on hazelnut samples would surely be very beneficial for trying to improve the performances of the SCiO portable spectrometer, as well as to further improve, test, and validate the performances of the MPA instrument in both acquisition modes, hopefully leading to more robust models (especially regarding the obtained RPD values). Such improvements could be very useful to move the present study to a higher development stage of rapid and cost-effective in situ analyses, which should be aimed at reaching, at least, a robust classification as “quality control”, according to the framework described by Williams.

## Figures and Tables

**Table 1:** Regression parameters from PLS models for the three NIR techniques. The three models have different complexities (number of latent variables, LVs), and the parameters used for describing the performances are the coefficient of determination (R^2^) and the root mean squared errors (RMSEs). The subscripts stand for CAL = calibration, CV = cross validation, and PRED = prediction (test set).

	SCiO	MPA Sphere	MPA Probe
LVs	7	4	3
R^2^_CAL_	0.717	0.925	0.846
R^2^_CV_	0.461	0.903	0.793
R^2^_PRED_	0.550	0.713	0.897
RMSE_CAL_	0.966	0.566	0.870
RMSE_CV_	1.332	0.645	1.008
RMSE_PRED_	1.217	1.109	0.712
RPD_CAL_	1.904	3.709	2.583
RPD_CV_	1.380	3.254	2.229
RPD_PRED_	1.909	1.773	2.167

**Table 2:** Interpretation of spectroscopic signals related to the fatty acids, with literature references.

	SCiO	MPA (Sphere and Probe)
C–H [30,31,32]	920 nm, stretching third overtones	1214 nm, second overtone CH_2_ 1390 nm, stretching CH_2_ 1730–1760 nm, first overtone 2300–2340 nm, stretching and deformation combinations CH_2_
C=C [30,31,32]	/	2100 nm, stretching
O–H [33]	960–1050 nm, stretching second overtones	1900 nm, combination band
Unassigned	780 nm, 830–850 nm	1600 nm

## Data Availability

The data presented in this study are available on request from the corresponding author.
